# Plague: Infections of Companion Animals and Opportunities for Intervention

**DOI:** 10.3390/anil020242

**Published:** 2011-06-21

**Authors:** Petra C.F. Oyston, Diane Williamson

**Affiliations:** Biomedical Sciences, Dstl Porton Down, Salisbury, Wiltshire, SP4 0JQ, UK; E-Mail: dewilliamson@dstl.gov.uk

**Keywords:** plague, vaccine, cats, dogs, fleas

## Abstract

**Simple Summary:**

Plague is a notorious disease of humans, typically transmitted from rodents to man by the bite of infected fleas. However, plague can also be brought into the home by domestic animals. Cats are acutely susceptible to plague and can pose a significant hazard to close contacts. Dogs are relatively resistant to plague, but can import infected fleas into the home. This review discusses options available for vaccinating cats and dogs, to protect the animals, their owners and veterinarians from infection.

**Abstract:**

Plague is a zoonotic disease, normally circulating in rodent populations, transmitted to humans most commonly through the bite of an infected flea vector. Secondary infection of the lungs results in generation of infectious aerosols, which pose a significant hazard to close contacts. In enzootic areas, plague infections have been reported in owners and veterinarians who come into contact with infected pets. Dogs are relatively resistant, but can import infected fleas into the home. Cats are acutely susceptible, and can present a direct hazard to health. Reducing roaming and hunting behaviours, combined with flea control measures go some way to reducing the risk to humans. Various vaccine formulations have been developed which may be suitable to protect companion animals from contracting plague, and thus preventing onward transmission to man. Since transmission has resulted in a number of fatal cases of plague, the vaccination of domestic animals such as cats would seem a low cost strategy for reducing the risk of infection by this serious disease in enzootic regions.

## 1. Yersinia pestis

Plague is caused by the bacterial pathogen *Yersinia pestis*, a Gram-negative non-motile, non-spore-forming coccobacillus. It is capable of growth between 4 and 40 °C, but grows optimally at 28-30 °C. Growth is somewhat slow, requiring 48 h on rich media for colony formation. The organism exhibits a range of nutritional requirements, having mutated genes in many metabolic pathways [1,2].

*Y. pestis* is a clonal derivative of the enteropathogen *Yersinia pseudotuberculosis* estimated to have emerged relatively recently in evolutionary terms, between 1,500 and 20,000 years ago [3]. Historically, three biovars have been recognised, Antiqua, Medievalis and Orientalis, differentiated by their ability to ferment glycerol and to reduce nitrate. Strains belonging to all the biovars are virulent and it has been suggested that they are linked to separate pandemics. Nowadays biovar Antiqua strains are isolated in Africa, and may be descended from the bacteria that caused the first pandemic. Medievalis strains are isolated in Central Asia and may be related to the bacteria of the second pandemic. Orientalis strains are widespread and appear to be the cause of the current pandemic [4]. However, modern taxonomic methods have shown the nomenclature to be more complicated than originally described (reviewed by [5]), and many researchers now favour the inclusion of a fourth biovar, Pestoides, first used for strains isolated from vole populations in the Former Soviet Union. However, despite the advent of modern molecular taxonomy methods e.g., [6], the inherent variability of *Y. pestis* strains still provokes discussion as to the best way to divide the species (reviewed by [5]).

A comparison of the *Y. pestis* and *Y. pseudotuberculosis* genomes reveals numerous genetic differences reflecting differences in virulence and niche [7]. In the transition from an enteropathogen found widely in the environment to an arthropod-vectored systemic pathogen, genes have been acquired, inactivated and rearranged [7,8]. The first plague genome to be elucidated was from a biovar Orientalis strain, CO92 [8], followed subsequently by the sequence of a biovar Medievalis strain, KIM [9]. The genome of strain C092 consisted of a 4.56 Mb chromosome while that of KIM was 4.6 Kb. In addition to the 75 Kb *Yersinia* virulence plasmid pYV/pCD1 which it possesses in common with *Y. pseudotuberculosis* and *Y. enterocolitica*, two further plasmids are possessed uniquely by *Y. pestis*, pFra/pMT1 (100–110 Kb), and pPst/pPCP/pPla (9.5 Kb) [10-12], which carry many of the exclusive virulence factors of *Y. pestis*. The genome possesses a large number of insertion sequences and appears to have undergone frequent intragenomic recombination. Indeed, recombination appears to be an on-going process even in the present day [8]. Complete genome sequences are available for many strains of *Y. pestis*. For example, a recent study compared the complete genome sequences of 17 strains [6], and these comparisons are informing on the microevolution and history of plague. Whilst the organism has acquired additional genes during its adaptation from enteric pathogen to systemic, insect-vectored pathogen, the bacterium has also many pseudogenes in pathways no longer essential in its new niche. Many pseudogenes have been identified in the genomes of fully virulent *Y. pestis* strains indicating that genome reduction processes are underway. *Y. pestis* appears to have passed through an evolutionary bottleneck and in its relatively isolated niche is unable to restore the array of pseudogenes it now possesses. It is thus probably in an evolutionary dead-end.

## 2. Plague

Plague occurs principally in three clinical forms in humans: bubonic, septicaemic and pneumonic plague. Other, uncommon presentations of plague include pharyngeal/enteric plague and meningeal plague. Pharyngeal/enteric plague can arise as a result of exposure to infectious aerosols or by ingestion of infected meat [13].

Bubonic plague is the classical and most common form of the disease. Bubonic plague arises as a result of the bite from an infected flea or by contamination of an open skin lesion. Upon infection, bacteria are phagocytosed and carried to the local lymph nodes draining the site of infection where they multiply. In humans, the resulting swollen, tender, inflamed lymph node is termed a bubo. The bacteria lyse the phagocytic cells and enter the bloodstream and are taken up by cells of the liver and spleen. Multiplication in these organs can result in populations of a million bacteria per gram of tissue [1]. Bacteria appear in ever increasing numbers in the bloodstream until death, probably resulting from endotoxic shock. Patients present with fever, chills, headache and a painful bubo. Local bacterial proliferation is sometimes evident in the form of an abscess or ulcer at the site of infection. Symptoms of fever and malaise develop 2-6 days post-infection, although the bubo may not be evident early in infection, and deeper lymph nodes may not be visible as lymphadenopathy develops. Patients may develop a significant bacteraemia. Those with blood colony counts higher than 100/mL have higher fatality rates, although a patient with 10^7^/mL did survive [14]. Untreated, the case fatality rate is 40-60%, but where therapy is used this can be reduced to around 14% [15]. Secondary pneumonia, arising from spread to the lungs, occurs in approximately 10% of cases, and these patients pose a significant risk to contacts due to production of infectious aerosols, and as such quarantine is recommended.

When *Y. pestis* infection with bacteraemia occurs without the development of lymphadenopathy, this is primary septicaemic plague which occurs in about 10–25% of cases [16,17]. Symptoms of septicaemic plague resemble those of most Gram negative septicaemic infections: fever, chills, headache, malaise. Due to difficulties in diagnosis and thus delays in appropriate antibiotic therapy, mortality rates are higher than for bubonic plague. Untreated, septicaemic plague is almost always fatal.

Human epidemic spread primarily occurs when a plague victim develops secondary pneumonia. The highly infectious respiratory aerosols allow the development of a primary pneumonic plague epidemic, which is of concern in public health. Primary pneumonic plague arises as a result of inhalation of plague bacilli in infectious aerosols, such as would be produced when there are pneumonia complications in bubonic plague. There is a short incubation period of 1-3 days, after which there is sudden onset of flu-like symptoms including fever, chills, headache, generalised body pains, weakness and chest discomfort. A cough develops with sputum production, which may be bloody, and increasing chest pain and difficulty in breathing. Pneumonic plague is invariably fatal unless antibiotic therapy commences within 24 h of the development of clinical symptoms [18].

Death occurs as a result of shock, probably due to endotoxin, resulting in disseminated intravascular coagulation, multiple organ failure and respiratory distress syndrome. Disseminated intravascular coagulation can lead to arteriolar thrombosis, haemorrhage in the skin and organs, and sometimes results in acral cyanosis and tissue necrosis. In addition to potential complications of meningitis and pneumonia, there may be generalized lymphadenopathy and abscess formation on the liver and spleen.

Plague is primarily a zoonotic disease affecting rodents. A range of other mammals is susceptible to infection, but play no role in the long-term survival of the organism. The susceptibility of different animal species varies. Humans are highly susceptible, while rodents which form the enzootic hosts are more resistant, although the susceptibility of different rodent species may vary. Transmission between rodents is by infected fleas and the cyclic infection of rodent and flea is essential for the maintenance of plague in nature. Fleas become infected upon feeding on the blood of an infected rodent suffering from septicaemic plague. The bacteria are restricted to the alimentary tract of the flea where they multiply in the midgut. The bacteria form large brown clumps which extend throughout the midgut, oesophagus and proventriculus, a valve-like chamber situated between the oesophagus and the midgut. The clumps increase in mass over a few days until they block the proventriculus and the flea becomes “blocked”. The flea thus begins to starve. As it futilely sucks blood from the host, the blood meal mixed with bacteria from the foregut is regurgitated into the mammal. Blockage of the flea, and thus efficient transmission of bacteria, has been shown to be dependent on the *Y. pestis* hemin storage locus (hms) [19]. Although originally identified as being responsible for storage of hemin in the outer membrane, the hms locus appears to be required for the hydrophobic surface properties of the bacterium. *Y. pestis* hms mutants are hydrophilic and thus do not autoaggregate in aqueous environments. Thus they are unable to colonize the proventriculus and produce blockage, although they do colonize the midgut and produce the large pigmented biofilm masses. Biofilm formation in the flea has been shown to be regulated by levels of c-di-GMP [20]. These masses are not absolutely required for transmission, but rather seem to be important in maintaining infection in the flea [21]. A phospholipase D, previously designated Yersinia murine toxin, has also been shown to be important in *Y. pestis* survival in the flea gut by protecting the bacterium from antimicrobial products in the blood meal [22]. However, while *Y. pseudotuberculosis* possesses many of the genes implicated in biofilm formation, such as hms, it is unable to form the biofilm in the flea [23]. Additionally, *Y. pseudotuberculosis* is acutely toxic to fleas compared to *Y. pestis* indicating that *Y. pestis* has modified its virulence for the vector to facilitate transmission [24]. The determinants carried by pPst/pPCP/pPla, but not pYV, have been implicated in flea blockage, but a strain of plague cured of the plasmid carrying the gene encoding Pla was able to block fleas normally [25], indicating that the procoagulant ability of Pla does not mediate blockage. Natural isolates and mutants defective for F1 capsule expression retain virulence, although the effects were influenced by the host [14,26]. However, recently it was reported that a mutant defective in F1 capsule production showed reduced transmission from the infected flea to the host, despite no observed defect in flea colonisation [27]. Thus the increased transmissibility of encapsulated strains would explain why the majority of clinical isolates express F1, but how F1-defective strains still occasionally cause disease under conditions of high flea burdens.

Flea-vectored transmission depends upon a significant septicaemia developing in the infected mammal. The Oriental rat flea *Xenopsylla cheopis* is considered the classic vector with the highest transmission efficiency of fleas studied, while cat fleas *(Ctenocephalides felis)* and human fleas *(Pulex irritans)* were found to be poor vectors [28,29]. However, this dogma is being challenged by new studies into vector competence and transmission by unblocked fleas (reviewed by [30]). Cat fleas were shown to be the most prevalent fleas in the home in a study in Uganda [31], and human fleas were often found in houses in Tanzania [32], and although they are less efficient at transmitting plague than rat fleas, their close proximity and diversity of hosts in which they are willing to feed, means they may pose a threat to humans. Although transmission to humans is usually through the bite of an infected animal flea, human-to-human transmission by fleas is considered rare, and the role in *P. irritans* in such transmission remains unproven (reviewed by [32]).

## 3. Infection of Companion Animals

Over 200 species of mammal have been reported to be able to become infected with *Y. pestis*. Of these, rodents are the most important hosts for plague in enzootic regions. Death of large numbers of rodents is one indication that plague is erupting in the local animal population. For example, rat deaths in large numbers were seen prior to the outbreak of bubonic plague in India in 1994 [33]. Serological surveillance of animals is another method of monitoring plague activity in a given area. Texas, California, Colorado and New Mexico all have on-going surveillance programmes for plague infection of rodents and carnivores. Insecticides must be used to kill fleas if rodent hosts are to be killed, and this must be done before rodenticides are employed. Such approaches are labour-intensive and not particularly effective on a large scale in enzootic areas. More effective are measures to eliminate habitat for rodents and reduce the appeal of residential areas to rodents, combined with treatment of domestic pets for fleas [34]. However, during an outbreak of plague in humans it is important to control populations of both fleas and rodents. If the rodent population has been reduced in number by plague, fleas will seek alternative hosts, including man, resulting in spread of bubonic plague. Thus flea populations must be reduced before control of rodent reservoirs can then be undertaken. Safe disposal of rodent corpses is a further priority in rodent control. Rural areas pose a specific problem in rodent control as removal of rodents from around habitation can result in subsequent invasion by field rodents. Therefore, rodent-proofing to prevent re-entry is important.

In enzootic areas, carnivorous companion animals, such as domestic cats and dogs, are a potential source of infection for human contacts. Domestic animals can become infected by ingesting infected rodents or through flea bites. Dogs present with a relatively mild disease, mainly non-specific fever and lethargy [35], and do not directly transmit plague to humans. However, rodent fleas living on dogs can transmit *Y. pestis* to humans [17,36,37], and allowing dogs in enzootic areas to sleep on the same bed as members of the household was a statistically significant risk factor for infection [37]. Therefore dogs should be treated for fleas, especially during the peak plague season (April to October in the US), and deterred from sleeping on beds, as such extended contact significantly increases the risk of infection. Veterinarians should also provide similar general advice to dog owners, but as plague in dogs is relatively mild and self-limiting it is unlikely practitioners will see anything but a small minority of canine cases [37]. Dogs develop antibody titres to F1 antigen after exposure, and as such have been suggested as a sentinel for plague monitoring [38].

In contrast to dogs, cats are highly susceptible to plague and can transmit the organism directly to humans [39]. By transdermal or oral routes, animals rapidly developed an acute febrile illness [38]. Cats fed infected rodents maintained *Y. pestis* in the throat for at least 10 days, but not if infected subcutaneously. Possibly linked to this, transmission by the bite of an infected kitten has been reported [40]. Cats also developed large abscesses with a tendency to rupture, releasing a purulent discharge containing large numbers of viable bacilli [38]. Although this discharge poses a contact hazard, another significant hazard is posed by aerosol transmission to humans to cause pneumonic plague. In a study of 60 cats with plague, 8 were found to be pneumonic: the owner of one of these pneumonic cats subsequently died of pneumonic plague [41]. The peak month for feline plague was found to be July, with cases being reported from March to November. Close contact with cats in enzootic regions is an important risk factor for human plague infections, and veterinarians are at particular risk: in an analysis of cases in New Mexico, of 10 cat-associated plague infections, 4 of the individuals were veterinarians (reviewed by [41]). Control measures to reduce the risk of cat-associated plague include treatment of animals for fleas, neutering of males to reduce roaming and the wearing of masks and gloves when handling live or dead cats suspected of infection in enzootic areas. Cats develop an antibody response to the F1 antigen upon infection, which may be useful for triggering human prophylaxis.

## 4. Prospects for Vaccination

Killed whole cell vaccines (KWCVs) for plague have been available since the late 1800's, albeit in different formulations [42]. The currently available KWCV is produced by the Central Serum Laboratories (CSL, Victoria, Australia) and comprises a suspension of heat-killed organisms (>10^9^/mL). Whilst epidemiological evidence suggests that such a KWCV is efficacious against bubonic plague [43], it also suggests that this formulation has little protective efficacy against pneumonic plague [1,44,45]. Furthermore, KWCVs do not protect against F1 negative strains of *Y. pestis*, suggesting that F1 is the key protective antigen in KWCV formulations [46,47]. Indeed, mice passively transfused with 500 μg IgG taken from guinea-pigs immunised with a KWCV supplemented with V-antigen were better protected against bubonic plague than mice transfused with guinea-pig IgG taken from animals immunised with either KWCV alone or the combination of F1 and V protective antigens [48].

On the other hand, live attenuated vaccines such as the EV series (e.g., EV76) are protective against pneumonic plague in murine [49] and macaque [50] models and have been used in man, particularly in Asia, Africa and Eastern Europe (reviewed by [51]). Recently, a live attenuated strain of *Y. pestis* KIM has been proposed as a candidate vaccine for plague, protecting 80% of vaccinated mice against pneumonic plague [52]. This differential in efficacy against pneumonic plague between live attenuated and killed vaccines has been attributed to the lack of the V antigen in the KWCV formulations which contain effective quantities of the F1 antigen only [45,53]; by comparison, live attenuated vaccines contain both F1 and V antigens [53]. However, live attenuated vaccines such as EV76, have caused morbidity in non-human primates [54] and are highly reactogenic in humans (reviewed by [51]), raising safety concerns over their use in man. In this context, there has been renewed research and development effort in the last 20 years towards alternative live attenuated and sub-unit vaccines with efficacy against pneumonic plague.

*Y. pestis* produces a range of antigens and virulence factors which have potential as sub-unit vaccine candidates (reviewed by [55]) and from this range, three proteins have been found to be promising vaccine candidates: F1 antigen, V antigen, and *Yersinia* secretory factor F (YscF). All of these proteins are virulence factors in *Y. pestis*: as mentioned above, F1 antigen is a capsular protein; V antigen and YscF are components of a Type three secretion system (TTSS). V antigen is found both within the bacterium, where it has a regulatory function, and also at the tip of the injectisome [56], whereas YscF is the main component of the *Yersinia* TTSS injectosome needle [57]. Other proteins have also been evaluated as vaccine antigens, such as Pla described above. However, Pla was found to be poorly immunogenic and provided no protection against lethal plague in a mouse model [58,59].

Whilst YscF conferred partial protective efficacy in a murine model of plague [60], full protective efficacy against *Y. pestis* was achieved with either F1 or V and maximised by combining these either in free association, or as a genetic fusion [47,53]. The protective efficacy of recombinant F1 and V subunits against *Y. pestis* has been reported by a number of laboratories and in a range of laboratory animal models including guinea pigs [48,61], rats [62] and non-human primates [50,63]. Vaccination with these sub-units has been shown to protect animal models against flea-vectored plague [64] as well as against experimental *Y. pestis* challenges, including pneumonic infections [45,62,63,65], unlike KWCVs. The F1 and V antigens are also immunogenic in humans [66]. The two antigens have been expressed as a genetic fusion in which the C-terminus of the F1 antigen is fused to the N-terminus of the V-antigen. This recombinant F1-V fusion has similar protective efficacy against pneumonic plague in the mouse and macaque as do the combined antigens [47,67,68], and mice were protected against *Y. pestis* by the passive transfer of immune serum from immunised macaques or humans [69]. The protection demonstrated by the F1 and V antigen vaccines against pneumonic plague is a significant advance compared to KWCV formulations, which have not been demonstrated to fully protect against this form of the disease.

Alternative formulations and delivery routes of the F1 and V proteins are also being explored and these may be useful for veterinary application. With a view to oral administration of the vaccine, the F1-V fusion has been expressed in transgenic plants and fruit and was demonstrated to be immunogenic by oral or sub-cutaneous delivery [70-72], and the expression of the F1 and V antigens by attenuated Salmonella strains dosed orally has been demonstrated to protect mice from bubonic plague [73,74]. Oral immunisation of mice with F1 and V antigens formulated in an amphipathic oily emulsion [75], or in cationic liposome-nucleic acid complexes (CLDC) combined with F1 antigen, has also been shown to protect mice partially or fully against pneumonic plague [76].

Encapsulation of the F1 and V proteins in biodegradable polymeric micro- and nano-spheres has been use to immunise mice by needle-free routes. Such formulations were shown to induce protective immunity against bubonic and pneumonic plague in the mouse after only a single immunisation [77] and this approach may be particularly suited for the prophylactic immunisation of domestic animals. The F1-V fusion, similarly formulated in polyanhydride nanoparticles for intranasal delivery, protected mice against subsequent intranasal challenge with *Y. pestis* [78] or in a proteosome adjuvant, protected them against aerosol challenge [79]. Trans-cutaneous delivery has also been used with the F1 and V proteins or the F1-V fusion to protect mice against plague [80,81], although several applications were required to induce protection [80].

Although these experimental vaccines have been developed primarily to prevent plague in man, the immunisation of domestic animals with some of these formulations could similarly be used to protect them against bubonic plague caused by infected flea bite. This would reduce morbidity in companion animals, and the risk of transmission to other animals or man. Sub-unit vaccines are regarded as the safest type of vaccine, and this is the approach being taken for licensing of the next generation human vaccine. However, live attenuated vaccines may also be a possibility for veterinary application, if regulatory and safety requirements can be fully satisfied. Empirically derived attenuated strains have been identified as described above. For example, the EV76 strain is partly attenuated by spontaneous deletion of the pigmentation locus. However, further defined attenuation would be required to improve safety and environmental impact profiles. The rational attenuation of *Y. pestis* has been demonstrated through a range of mutations [82-86], or by modifying the interaction of the pathogen with the host immune system [52]. Of particular note in this context, is the fatal case of septicemic plague reported in a researcher handling the pigmentation-negative *Y. pestis* KIM D27 strain: this strain is highly attenuated in mice, and regarded as relatively safe to handle. Post-mortem analyses identified this individual to have previously undiagnosed hereditary hemochromatosis, causing an iron overload in his blood and tissues; this is thought to have compensated for the iron-acquisition defect of KIM D27 resulting in restoration of virulence [87]. Additionally, this individual had insulin-dependent diabetes mellitus, a further risk factor for susceptibility to microbial infection. This case illustrates the caution required when considering a live attenuated strain of *Y. pestis* for human use as a vaccine and such caution should be extended to veterinary use where environmental escape is possible.

Rodents are amplifying hosts for plague and thus a source of infection for domestic animals. Thus, controlling plague in wild rodents is an alternative way to prevent infection in domestic animals. Therefore, the vaccination of feral species has been evaluated. A live vaccine vector comprising a Raccoon Pox vaccine expressing F1 and a truncated V antigen was formulated into bait for oral vaccination of black-tailed prairie dogs [88]. This vaccine induced protective immunity against plague challenge and indeed was more protective than two doses of injected F1-V adsorbed to alum [88]. Similarly, the F1-V antigen fusion was used to immunise black-footed ferrets, an endangered species being reintroduced to Montana, and was demonstrated to enhance protection, particularly in the absence of flea control measures [89]. Other experimental vaccines, such as a Vaccinia Virus expressing the F1-V fusion [90] or recombinant Salmonella expressing similar antigens (reviewed by [91]), have been described and suggested as suitable for immunisation of animals known to be reservoirs in enzootic regions.

Thus a number of vaccination options exist for protecting companion animals in endemic areas from contracting plague via infected fleas, and thus preventing onward transmission to man. Since transmission has resulted in a number of fatal cases of plague, the vaccination of domestic animals such as cats would seem a low cost strategy for reducing the risk of infection by this serious disease in enzootic regions.

## Declaration

© Crown copyright Dstl 2011.
